# Oregano Essential Oil Improves Intestinal Morphology and Expression of Tight Junction Proteins Associated with Modulation of Selected Intestinal Bacteria and Immune Status in a Pig Model

**DOI:** 10.1155/2016/5436738

**Published:** 2016-05-29

**Authors:** Yi Zou, Quanhang Xiang, Jun Wang, Jian Peng, Hongkui Wei

**Affiliations:** ^1^Department of Animal Nutrition and Feed Science, College of Animal Science and Technology, Huazhong Agricultural University, Wuhan 430070, China; ^2^The Cooperative Innovation Center for Sustainable Pig Production, Wuhan 430070, China

## Abstract

Oregano essential oil (OEO) has long been used to improve the health of animals, particularly the health of intestine, which is generally attributed to its antimicrobial and anti-inflammatory effects. However, how OEO acts in the intestine of pig is still unclear. This study was aimed at elucidating how OEO promotes the intestinal barrier integrity in a pig model. Pigs were fed a control diet alone or one supplemented with 25 mg/kg of OEO for 4 weeks. The OEO-treated pigs showed decreased (*P* < 0.05) endotoxin level in serum and increased (*P* < 0.05) villus height and expression of occludin and zonula occludens-1 (ZO-1) in the jejunum. These results demonstrated that the integrity of intestinal barrier was improved by OEO treatment. The OEO-treated pigs had a lower (*P* < 0.05) population of* Escherichia coli* in the jejunum, ileum, and colon than the control. This is in accordance with the greater inactivation (*P* < 0.05) of inflammation, which was reflected by the mitogen-activated protein kinase (MAPK), protein kinase B (Akt), and nuclear factor *κ*B (NF-*κ*B) signaling pathways and expression of inflammatory cytokines in the jejunum. Our results show that OEO promotes intestinal barrier integrity, probably through modulating intestinal bacteria and immune status in pigs.

## 1. Introduction

The ability of the intestinal epithelium to function as a barrier between the external environment and the closely regulated internal milieu is essential for human and pig health [1, 2]. Increased intestinal permeability is a potential factor of gastrointestinal dysfunction and pathology, including Crohn's disease, multiple organ dysfunction, bacterial translocation, food allergies, and acute pancreatitis [3, 4]. Currently, antibiotics are widely available and have a variety of proposed beneficial effects to promote intestinal health, but the application of these drugs is limited by their toxicity and side effects [5]. Therefore, it is urgent to find alternative treatments with fewer side effects.

Oregano (*Origanum vulgare* L.) is an aromatic plant widely distributed throughout the Mediterranean area and Asia [6]. Oregano essential oil (OEO), a volatile oil, is concentrated from natural plant products which contain the volatile aroma compounds. These mixtures of volatile compounds exert different biological actions, such as antimicrobial, anti-inflammatory, and antioxidative activities [7]. There have been evidences showing the therapeutic effects of OEO supplementation on barrier defects in the gut of mammals, including mouse, rat, and broiler models [8–10]. However, there has been no report about the use of OEO supplementation to improve the intestinal barrier integrity of pigs.

Several studies have indicated that intestinal microbiota and immune status are important factors that influence the function of the intestinal barrier [11]. Alteration of the microbial composition results in increased immune stimulation, epithelial dysfunction, or enhanced mucosal permeability [12]. In the present study, we hypothesized that dietary OEO supplementation promotes intestinal barrier integrity by regulating intestinal bacteria and inflammation. We thus would use the pig model to test this hypothesis and study the morphology and permeability of the intestine, the composition of the intestinal microbiota, the activation of innate immunity, and the expression of proinflammatory cytokines after OEO supplementation.

## 2. Materials and Methods

All animal handling protocols were approved by the Huazhong Agricultural University Animal Care and Use Committee guidelines.

### 2.1. Animals, Diets, and Treatments

A total of 170 pigs (Large White × Landrace) with an initial body weight (BW) of 72 kg (±4.0 kg) were obtained from the same farm (Wuhan China Pork Co. Ltd., Wuhan, China). The pens were located in a building at a temperature maintained between 15 and 25°C. For the experiment, the 170 pigs were split into two groups according to the diet (each group of 85 pigs was further split into 5 replicate pens each holding 17 pigs): (1) control treatment without supplementation and (2) supplementation of OEO (25 mg/kg of feed for 28 d, as-fed basis). The composition of the control diet is shown in Table 1. The OEO was in the form of a powder called Phytogen (Meritech Bioengineering Co. Ltd., Guangzhou, China). The components of OEO are shown in Supplementary Table S1 (see Supplementary Material available online at http://dx.doi.org/10.1155/2016/5436738). Pigs were allowed feed and water ad libitum over a period of 4 weeks.

### 2.2. Sample Collection

On the day of slaughter, between 9:00 and 14:00, a total of 12 pigs (100 kg BW), with 6 pigs from each dietary treatment, were transported in an open truck to the slaughterhouse. Blood samples were collected after electrical stunning and then quickly separated into five tubes. A 10 mL sample was placed on ice immediately, which was subsequently centrifuged at 1300 ×g at 4°C for 15 min to obtain serum. The serum samples were stored at −80°C for subsequent analysis. The digesta samples were immediately removed from the jejunum, ileum, and colon of each pig and stored at −80°C until further analysis. Samples of the jejunum itself were removed from the middle jejunum segment and then rinsed with ice-cold physiological saline. One section was snap-frozen in liquid nitrogen and then stored at −80°C until further analysis. Other sections of jejunum (3 cm) were kept in 4% neutral buffered formalin for gut morphological analysis.

### 2.3. Gut Morphological Analysis

The digestive tract was removed from the jejunum and fixed in 10% phosphate-buffered formalin. The samples were sectioned at 5 mm thickness and stained with hematoxylin and eosin. Villus height, villus width, and villus crypt depth were measured on the stained sections using a light microscope fitted with an image analyzer (Image Pro Plus 6.0, Media Cybernetics, Bethesda, MD, USA). Twenty villi and crypts were measured for each segment.

### 2.4. Measurement of Serum Endotoxin Level

Serum endotoxin level was measured by a quantitative chromogenic end point tachypleus amebocyte lysate endotoxin detection kit following the manufacturer's instructions (Xiamen TAL Experimental Plant Co., Ltd., China). Briefly, serum samples were diluted to 1 : 10 with water/Tris-HCl buffer. After centrifugation at 1270 ×g for 10 min, the supernatant was removed and incubated with limulus amebocyte lysate at 37°C for 10 min, followed by incubation with the provided chromogenic substance for 6 min. The absorbance at 545 nm was measured after the addition of appropriate reagents.

### 2.5. Extraction of Microbial DNA from the Gastrointestinal Tract Digesta

Total DNA of jejunum, ileum, and colon* digesta* was extracted and purified from gastrointestinal tract digesta using a QIAamp DNA Stool Kit (Qiagen, Germany) according to the manufacturer's instructions. DNA concentration was determined by spectrophotometry (NanoDrop).

### 2.6. Protein Immunoblot Analysis

Briefly, 100 mg of frozen tissue of jejunum was homogenized in 1 mL RIPA lysis buffer (with 1 mM PMSF and 10 *μ*L/mL phosphatase inhibitors). For nuclear p65 measurement, the nuclear fractions were isolated using the Nuclear/Cytosol Fractionation Kit (BestBio, China). Next, they were centrifuged at 12000 ×g at 4°C for 10 min and the supernatants were collected for assay. After the protein concentration was determined by a standard BCA protein assay, protein sample was loaded per lane and separated on SDS-PAGE. The target protein was then electrophoretically transferred to nitrocellulose membranes, which were blocked in TBST (5% nonfat milk, 10 mM Tris, 150 mM NaCl, and 0.05% Tween-20) for 2 h. Next, they were incubated with first antibodies, anti-Phospho-p38 MAPK (1 : 1000, Cell Signaling, USA), anti-Phospho-JNK (1 : 1000, Cell Signaling, USA), anti-Phospho-ERK1/2 (1 : 1000, Cell Signaling, USA), anti-Phospho-Akt (1 : 1000, Cell Signaling, USA), anti-p38 MAPK (1 : 1000, Affbiotech, USA), anti-JNK (1 : 1000, Affbiotech, USA), anti-ERK1/2 (1 : 1000, Affbiotech, USA), anti-Akt (1 : 1000, Affbiotech, USA), anti-ZO-1 (1 : 1000, Affbiotech, USA), anti-occludin (1 : 1000, Affbiotech, USA), anti-NF-*κ*B p65 (1 : 1000, Cell Signaling, USA), anti-PCNA (1 : 5000, BD Transduction Laboratories, San Diego, CA), or anti-actin antibodies (1 : 1000, Cell Signaling, USA) at 4°C overnight. After three washes with Tris-buffered saline containing 0.1% Tween-20, blots were incubated with the HRP-conjugated secondary antibodies, anti-rabbit IgG (1 : 15,000, Jackson ImmunoResearch, USA) or anti-mouse IgG (1 : 15,000, Jackson ImmunoResearch, USA), for 2 h and were washed again. Chemiluminescence detection was performed using the ECL reagent (Thermo Scientific, Rockford, IL, USA) according to the manufacturer's instructions. Specific bands were detected and were analyzed and quantified by Image J Software (NIH, Bethesda, MD, USA).

### 2.7. Quantitative PCR

Total RNA was extracted from samples of jejunum using Trizol Reagent (Invitrogen, USA) according to the manufacturer's instructions. The DNA obtained from the intestinal luminal content was used as the template to analyze intestinal bacteria. Primers (Table 2) used in this study were either synthesized according to our previous protocols or designed with Primer 5.0 according to pig gene sequences. Real-time PCR was performed according to our previous study [10]. The relative expression of genes in the treatment group was normalized based on the values of the control group.

### 2.8. Statistics

Statistical analysis was performed using Prism software (Prism 5.0, GraphPad Software, La Jolla, CA). Numbers (*n*) used for statistics are noted in the figures. All data were analyzed by *t*-test procedures of SAS (v 8.2, SAS Inst., Inc., Cary, NC). All the values were presented as means ± standard error of the mean (SEM), and those at *P* < 0.05 were considered significant.

## 3. Results

### 3.1. Morphology of the Jejunum

**Figure 1:**
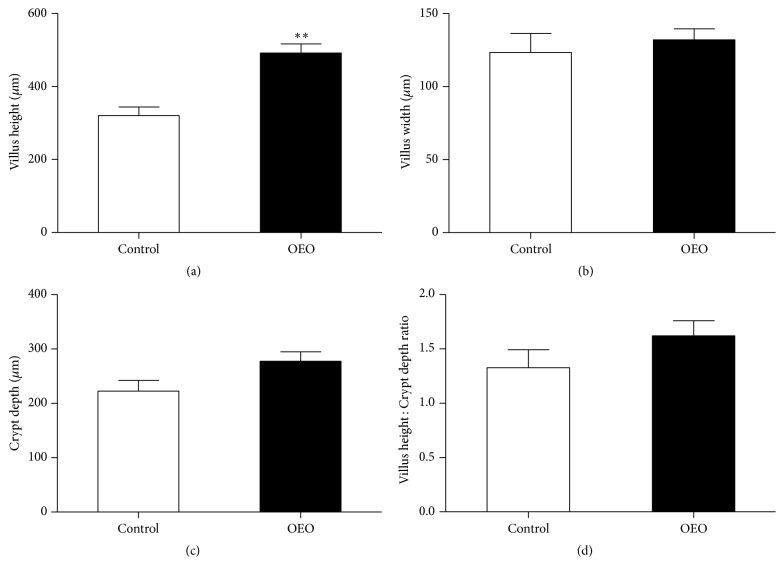
Effect of OEO on villus height, villus width, crypt depth, and the villus height : crypt depth ratio in the jejunum of pig. Values are means ± SEM, *n* = 6. ^*∗∗*^Significantly different (*P* < 0.01) from the control group.

**Figure 2:**
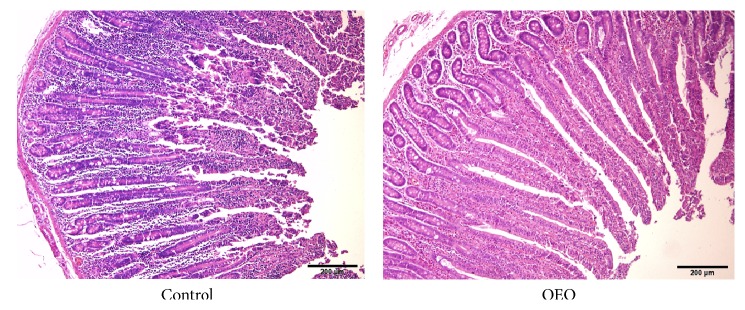
Effect of OEO on jejunum morphology in pig. The jejunum was cut off and fixed in 10% formaldehyde-phosphate buffer and then stained with hematoxylin and eosin. Hematoxylin and eosin staining with original magnification ×100. Bars represent 200 *μ*m.

Morphology is a good indicator of the status of the intestine [13]. As shown in Figure 1, although no significant differences were observed in villus width, crypt depth, and the ratio of villus height to crypt depth between different treatments (*P* > 0.05), the villus height of the OEO-treated pigs was significantly higher than that of the control (*P* < 0.05). As shown in Figure 2, the villi were scattered and seriously desquamated in the jejunum of the control group, while higher and intact villi were observed in the jejunum of OEO-treated pigs.

### 3.2. Endotoxin Levels in the Serum

**Figure 3:**
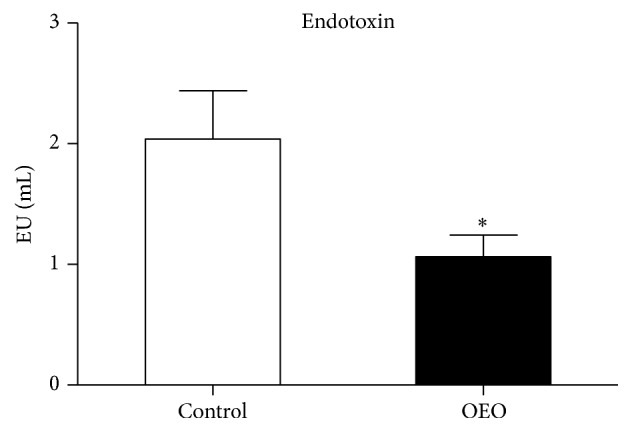
Effect of OEO on endotoxin levels in the serum of pig. Values are means ± SEM, *n* = 6. ^*∗*^Significantly different (*P* < 0.05) from the control group.

Endotoxin level is a useful biomarker for evaluating the integrity of the gastrointestinal tract [14]. The effects of OEO supplementation on endotoxin level in the serum of pigs are shown in Figure 3. The OEO-treated pigs showed a significantly lower (*P* < 0.05) concentration of endotoxin in the serum than the control.

### 3.3. The Expression of Barrier Tight Junction Proteins in the Jejunum

**Figure 4:**
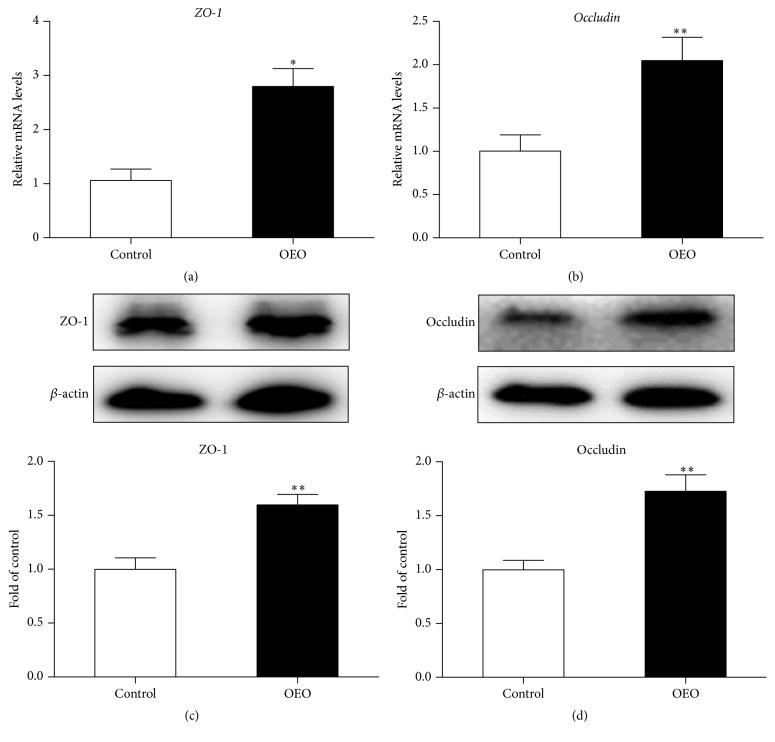
Effect of OEO on the ZO-1 and occludin levels in the jejunum of pig. (a)* ZO-1* mRNA level, (b)* occludin* mRNA level, (c) ZO-1 protein level, and (d) occludin protein level. Expression of the selected genes was quantified by quantitative reverse transcription-PCR. Equal loading was assessed by *β*-actin immunoblotting. Values are means ± SEM, *n* = 6. ^*∗*^Significantly different (*P* < 0.05) from the control group. ^*∗∗*^Significantly different (*P* < 0.01) from the control group. ZO-1, zonula occludens-1.

To determine the effects of OEO supplementation on the intestinal mucosal tight junction, the expression of occludin and zonula occludens-1 (ZO-1) was measured at mRNA and protein level. As shown in Figure 4, the mRNA levels of* occludin* and* ZO-1* were significantly higher in the OEO-treated pigs than in the control group (*P* < 0.01). Similarly, OEO supplementation also increased (*P* < 0.05) the abundance of occludin and ZO-1 protein in the jejunum.

### 3.4. Major Microbiota in Different Regions of the Intestinal Tract

**Figure 5:**
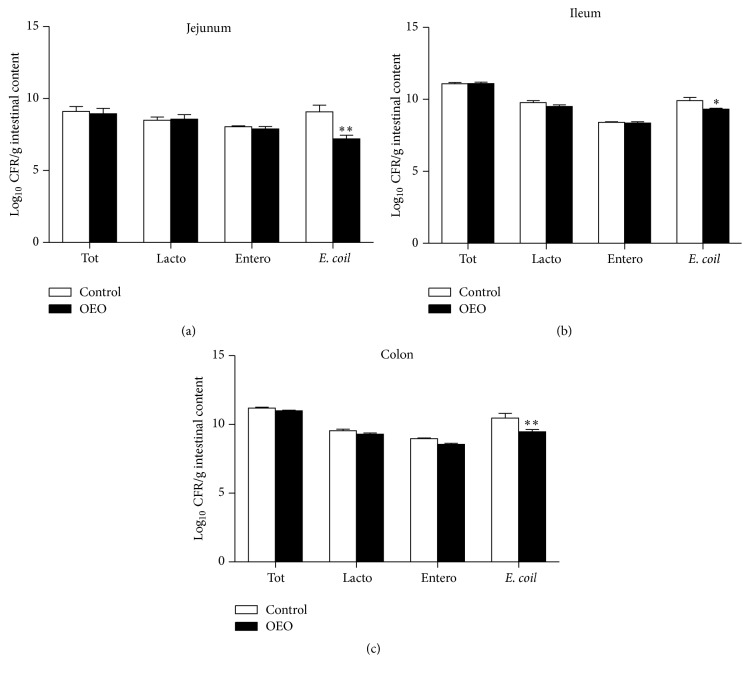
Effect of OEO on selected microbial populations in the jejunum, ileum, and colon tract of pig. Values are means ± SEM, *n* = 6. ^*∗*^Significantly different (*P* < 0.05) from the control group. ^*∗∗*^Significantly different (*P* < 0.01) from the control group. Tot, total bacteria; Lacto,* Lactobacillus* spp.; Entero,* Enterococcus faecalis*;* E. coli*,* Escherichia coli*; Log_10_, 16S rRNA gene copies/g contents.

The effects of OEO supplementation on selected microbial populations in different intestinal tracts are shown in Figure 5. Although there were no significant differences (*P* > 0.05) in the total bacteria,* Lactobacillus* and* Enterococcus* spp. populations, the OEO-treated pigs had a significantly lower population of* Escherichia coli* (*E. coli*) in the jejunum (*P* < 0.05), ileum (*P* < 0.05), and colon (*P* < 0.01) compared with the control.

### 3.5. MAPKs, Akt, and NF-*κ*B Pathways in the Jejunum

**Figure 6:**
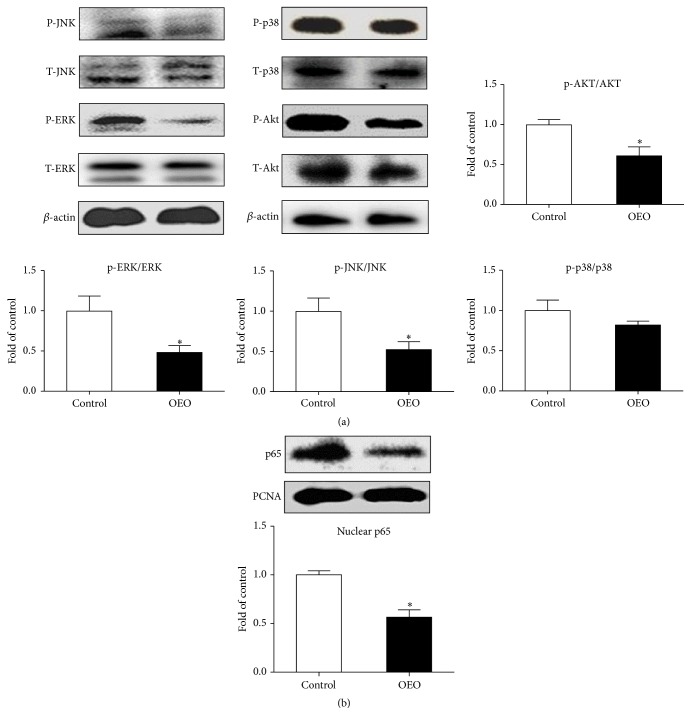
Effect of OEO on the levels of p-Akt, p-ERK, p-p38, p-JNK, and NF-*κ*B p65 proteins in the jejunum of pig. (a) Akt protein, ERK protein, p38 protein, and JNK protein levels. Equal loading was assessed by *β*-actin immunoblotting. (b) Nuclear p65 protein level. Equal loading was assessed by PCNA immunoblotting. Values are means ± SEM, *n* = 6. ^*∗*^Significantly different (*P* < 0.05) from the control group. ERK1/2, extracellular signal-regulated kinases 1/2; JNK, c-Jun N-terminal protein kinase; Akt, protein kinase B; NF-*κ*B, nuclear factor kappa B; P, phosphorylated; T, total.

To investigate the mechanism responsible for the protective effect of OEO against inflammation in the jejunum of pigs, we further studied the underlying signaling pathways: the MAPKs, Akt, and NF-*κ*B pathways, which are induced by intestinal microorganisms and have been shown to be implicated in the induction of proinflammatory genes [15]. As shown in Figure 6(a), in the jejunum, OEO supplementation inhibited (*P* < 0.05) the activation of JNK, ERK1/2, and Akt due to lower abundance of phosphorylated JNK, ERK1/2, and Akt proteins compared with the control group. Similarly, OEO supplementation decreased (*P* < 0.05) the abundance of NF-*κ*B p65 protein in the nucleus of the jejunum (Figure 6(b)). In contrast, OEO supplementation had no effect on phosphorylated p38.

### 3.6. The mRNA Levels of Proinflammatory Cytokines in the Jejunum

**Figure 7:**
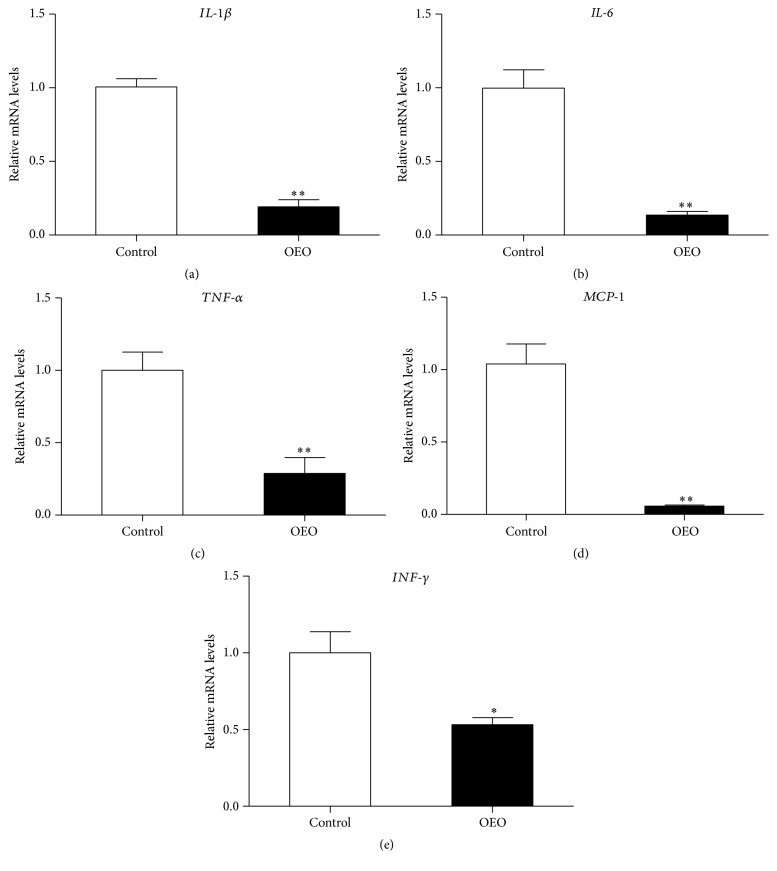
Effect of OEO on the mRNA levels of proinflammatory cytokines in the jejunum of pig. Expression of the selected genes was quantified by quantitative reverse transcription-PCR. (a)* IL-1β* mRNA level, (b)* IL-6* mRNA level, (c)* TNF-α* mRNA level, (d)* MCP-1* mRNA level, and (e)* INF-γ* mRNA level. Values are means ± SEM, *n* = 6. ^*∗*^Significantly different (*P* < 0.05) from the control group. ^*∗∗*^Significantly different (*P* < 0.01) from the control group. IL-1*β*, interleukin-1*β*; IL-6, interleukin-6; TNF-*α*, tumor necrosis factor-*α*; MCP-1, monocyte chemotactic protein-1; INF-*γ*, interferon gamma.

We examined the gene expression levels of four major inflammatory cytokines involved in mucosal inflammation in the jejunum tissues: interleukin-1*β* (IL-1*β*), interleukin-6 (IL-6), tumor necrosis factor-*α* (TNF-*α*), monocyte chemotactic protein-1 (MCP-1), and interferon gamma (INF-*γ*). The results are presented in Figure 7. The OEO-treated pigs showed significantly decreased (*P* < 0.05) levels of* TNF-α*,* IL-1β*,* IL-6*,* MCP-1*, and* INF-γ* compared with the control group.

## 4. Discussion

Defects in the intestinal barrier of animals can be induced by several types of ongoing environmental/life factors linked to physiology, psychology, antigens, and toxins [16]. Intestinal barrier defects are a serious problem in human as well as in pigs [17, 18]. Various aromatic plants and their products have been reported to have beneficial effects for the intestine of animals [19]. Furthermore, in a previous study of our laboratory, it was also observed that the intestinal barrier could be improved by OEO supplementation in rat models [10]. However, to our knowledge, there has been no report about the effect of OEO on the intestinal barrier of finishing pigs. Therefore, in the present study, we investigated whether dietary supplementation of OEO could have a protective effect on the intestinal barrier and whether OEO could be used as a feed additive for pigs.

Intestinal mucosal permeability is directly related to the integrity of the intestinal barrier [20]. The function of the intestinal barrier can be commonly assessed by many indexes, such as serum endotoxin level, intestine morphology, and intestinal tight junction proteins [14, 21, 22]. In the present study, the height of villi in the jejunum of pigs was increased after treatment with OEO, indicating that OEO may protect the intestine against villous atrophy and epithelial cell necrosis. Consistently, the endotoxin level in pig serum decreased significantly after treatment with OEO. These results were in agreement with previous findings, which demonstrated that using an OEO supplemented diet improves the ileum villus height and decreases serum endotoxin level in broilers [23]. In addition, we found that the expression of occludin and ZO-1, the two major tight junction proteins in epithelia affecting the organization and the stability of the tight junction [21], was significantly decreased by OEO treatment. Similarly, Placha et al. [9] also observed that broilers fed with OEO had improved intestinal barrier integrity. Our results indicate that OEO supplementation can be a promising approach for protecting the intestinal barrier in pigs.

The intestinal microbiota plays critical roles in the maintenance of mucosal homeostasis [11]. A greater population of* E. coli* might affect the intestinal mucosa because they release toxins, resulting in an intimate interaction between the microbiota and the host enterocytes [24, 25]. The abundance and composition of intestinal bacteria can be easily affected by various dietary factors [26]. In the present study, the dietary consumption of OEO decreased the populations of* E. coli* in the jejunum, ileum, and colon. These results are consistent with those of Tan et al. [27] and Sun et al. [23], who reported that that population of* E. coli* in intestinal is decreased in OEO-treated pigs and broilers, respectively. Thymol and carvacrol, the main active components of essential oil derived from thyme and oregano [7], were also documented to inhibit the proliferation of* E. coli in vitro* [28].

Recently, it has been recognized that the gut microbiota can influence the immune function beyond the gut [26]. The relationship between the immune system and the commensal flora is a precarious one, and perturbations in immune or epithelial homeostasis can lead to gut inflammation [29]. The initial sensing between bacteria and the host occurs through pattern recognition receptors [30]. In the intestine, the activated pattern recognition receptors trigger intestinal immune responses through various downstream signal transductions, such as MAPKs (e.g., p38 MAPK, ERK1/2, and JNK), Akt, and NF-*κ*B [31, 32]. The Akt and MAPKs pathways modulate intestinal innate immunity through regulating the phosphorylation of inhibitory *κ*B kinases to activate the NF-*κ*B pathway [33]. The NF-*κ*B pathway, which is activated by intestinal microbes, plays an important role in activating host proinflammatory responses [34]. The activation of these pathways is associated with the increased expression of TNF-*α*, IL-1*β*, IL-6, MCP-1, and INF-*γ* [35, 36]. The innate immune system is activated as a defense mechanism and is generally beneficial [37]. However, if the inflammation is uncontrolled, the migration of innate immune cells such as neutrophils, macrophages, and dendritic cells into the target mucosal tissues will result in mucosal injury [32]. Regulating intestinal inflammation is of great significance for intestinal health [15, 38, 39]. OEO has been found to possess a significant anti-inflammatory effect* in vitro* and* in vivo* [8, 40]. The present study indicates that OEO inhibits the inflammation in the intestine and downregulates the expression of* TNF-α*,* IL-1β*,* IL-6*,* INF-γ, *and* MCP-1* in the jejunum. Similarly, it has been previously shown that OEO effectively reduces the production of proinflammatory cytokine and thereby attenuates the TNBS-induced colitis in mice [41]. As the major component of OEO, carvacrol was also reported to probably influence the release and/or synthesis of inflammatory mediators [41]. As the mechanisms mediating the suppressive effects of OEO on inflammation are still unclear, we can only speculate that there might be several potential modes of action based on our results. One possibility could be the influence of OEO on NF-*κ*B p65 and phosphorylated MAPKs (ERK1/2 and JNK) and Akt pathways, which can activate the expression of the genes involved in immune and inflammatory responses, such as* TNF-α*,* IL-1β*,* IL-6*,* INF-γ, *and* MCP-1*. On the other hand, intestinal microbial disorder (such as increased number of* E. coli*) is a potent inducer of intestinal inflammation. The present study has shown that OEO can inhibit intestinal* E. coli* in pigs.

## 5. Conclusion

In conclusion, our results indicate that dietary administration of OEO can reduce the production of proinflammatory cytokines and promote the integrity of the intestinal barrier in pigs. The protective effect of OEO on the intestine is associated with the decrease of intestinal* E. coli *population and the inactivation of the JNK, ERK1/2, Akt, and NF-*κ*B signaling pathways. These results will contribute to a better understanding of the possible mechanisms by which OEO promotes the integrity of the intestinal barrier in pigs.

## Supplementary Material

Oregano essential oils are very complex natural mixtures which can contain about 32 components at quite different concentrations. Carvacrol (81.92%) is the major components of the oregano essential oils.

## Figures and Tables

**Table 1:** Composition and analysis of the basal diet.

	Basal diet^*∗*^
*Composition (g/kg) *	
Wheat	380.00
Corn, grains	464.10
Soybean meal (46%)	89.00
Monocalcium phosphate	14.00
Limestone	7.00
Mycetes adsorbent	1.50
Antimildew agent	0.50
Salt	3.50
Soybean oil	20.00
Ethoxyquin	0.25
Probiotics	0.20
Y402 premix^†^	20.00

*Analysis* ^‡^	
Dry matter, DM (%)	86.80
Metabolism energy (MJ/kg)	13.20
Crude protein, CP (%)	13.90
Crude fiber (%)	2.80
Ash (%)	3.60
Fat (%)	4.30
Calcium (%)	0.60
Phosphorus (%)	0.60

^*∗*^Control group (C) was fed with the above basal diet, whereas the oregano essential oil (OEO) group consumed the basal diet supplemented with 25 mg/kg OEO.

^†^Premix contained per kg 10.5 g Fe, 1.4 g Cu, 8.5 g Zn, 4 g Mn, 7.5 mg Se, 30 mg I, 350 kIU of vitamin A, 40 kIU of vitamin D3, 1.5 kIU of vitamin E, 50 mg of vitamin K3, 50 mg of vitamin B1, 150 mg of vitamin B2, 100 mg of vitamin B6, 0.1 mg of vitamin B12, 86.4 g lysine, 17.5 g methionine, 25 g threonine, 4 g phytase, and 15 g choline (kIU: 1000 international units).

^‡^Metabolism energy was calculated from data provide by Feed Database in China (1999).

**Table 2:** Species and genus specific primers used for real-time PCR.

Gene	Primers (sense/antisense 5′-3′)	Size (bp)	Annealing temperature (°C)
Total bacteria	F: ACTCCTACGGGAGGCAGCAG R: ATTACCGCGGCTGCTGG	175	60
*Lactobacillus* spp.	F: CACCGCTACACATGGAG R: TGGAAGATTCCCTACTGCT	341	58
*Escherichia coli*	F: CATGCCGCGTGTATGAAGAA R: TTTGCTCATTGACGTTACCCG	96	60
*Enterococcus faecalis*	F: CCCTTATTGTTAGTTGCCATCATT R: ACAATGGGAAGTACAACGAGT	144	61
*TNF-α*	F: CACCACGCTCTTCTGCCTACTG R: TTGAGACGATGATCTGAGTCCTTGG	115	63
*MCP-1*	F: GTCCTTGCCCAGCCAGATG R: CGATGGTCTTGAAGATCACTGCT	148	60
*IL-1β*	F: AAAGGGGACTTGAAGAGAG R: CTGCTTGAGAGGTGCTGATGT	286	58
*IL-6*	F: AAGGTGATGCCACCTCAGAC R: TCTGCCAGTACCTCCTTGCT	151	60
*INF-γ*	F: GAGCCAAATTGTCTCCTTCTAC R: CGAAGTCATTCAGTTTCCCAG	140	61
*ZO-1*	F: GGCGCACGGCGAAGGTAATT R: CTATCAAACTCAGGAGGCGGCACT	405	60
*Occludin*	F: GGAGTGATTCGGATTCTGTCTATGCT R: CGCCTGGGCTGTTGGGTTGA	423	60
*β-actin*	F: CCAGGTCATCACCATCGG R: CCGTGTTGGCGTAGAGGT	158	60
