# Supplementary material for: Oregano Essential Oil Improves Intestinal Morphology and Expression of Tight Junction Proteins Associated with Modulation of Selected Intestinal Bacteria and Immune Status in a Pig Model
Source: Biomed Res Int. 2016 May 29;2016:5436738. doi: 10.1155/2016/5436738 (PMC4903144; doi:10.1155/2016/5436738)
Supplement: Supplementary file 1 — Oregano essential oils are very complex natural mixtures which can contain about 32 components at quite different concentrations. Carvacrol (81.92%) is the major components of the oregano essential oils. [file 5436738.f1.pdf]

Table S1 - Chemical composition of oregano essential oil.

Components [a]	Composition %
α-Thujene/α-Pinene	0.66
Camphene	0.09
β-Pinene	0.07
Sabinene	0.04
Myrcene	0.86
α-Phellandrene	0.08
α-Terpinene	0.58
Limonene	0.13
1,8-Cineole+β-phellandrene	0.09
β-Ocimene	0.07
γ-Terpinene	4.49
3-Octanone	0.07
ρ-Cymene	3.07
Terpinolene	0.04
3-Octanel	0.01
1-Octen-3-ol	0.24
Dimethyl styrene	0.01
Trans-Sabinene hydrate	0.10
Linalool	0.28
Cis-Sabinene hydrate	0.06
1-Terpilool	0.04

Terpine-4-ol	0.34
Carvacrol methyl ether	0.22
β-Caryophyllene	1.41
Dihydrocarvone	0.08
α-Humulene	0.14
α-Terpineol	0.16
Bomeol	0.30
β-Bisaholene	0.70
Caryophyllene oxide	0.14
Thymol	3.50
Carvacrol	81.92
Total	99.99

[a] The dates were provided by Meriden Animal Health Ltd.
